# *De Novo* Assembly of the Quorum-Sensing *Pandoraea* sp. Strain RB-44 Complete Genome Sequence Using PacBio Single-Molecule Real-Time Sequencing Technology

**DOI:** 10.1128/genomeA.00245-14

**Published:** 2014-04-03

**Authors:** Robson Ee, Yan-Lue Lim, Wai-Fong Yin, Kok-Gan Chan

**Affiliations:** Division of Genetics and Molecular Biology, Institute of Biological Sciences, Faculty of Science, University of Malaya, Kuala Lumpur, Malaysia

## Abstract

We report the first complete genome sequence of *Pandoraea* sp. strain RB-44, which was found to possess quorum-sensing properties. To the best of our knowledge, this is the first documentation of both a complete genome sequence and quorum-sensing properties of a *Pandoraea* species.

## GENOME ANNOUNCEMENT

Quorum sensing is a communication mechanism that is known to mediate cell-to-cell interaction between proteobacteria through the production and detection of diffusible autoinducer molecules known as *N*-acyl homoserine lactones (1–3). Various essential bacterial activities are coordinated by this mechanism, for instance, virulence, formation of biofilms, antibiotic synthesis, motility, and swarming activities (3–5). *Pandoraea* spp. have been reported to be cystic fibrosis clinical pathogens, but their role in pathogenicity is still largely unknown (6–9). Here, we present the first complete genome sequence of a quorum-sensing *Pandoraea* sp. strain, RB-44, which was isolated from an ex-landfill dumping ground.

Genomic DNA was extracted using the MasterPure DNA purification kit (Epicentre, Inc., Madison, WI, USA), while DNA quality was determined via a NanoDrop spectrophotometer (Thermo Scientiﬁc, Waltham, MA, USA) and a Qubit 2.0 ﬂuorometer (Life Technologies, Carlsbad, CA, USA). Pacific Biosciences RS II sequencing technology (Pacific Biosciences, Menlo Park, CA, USA) was used as the sequencing platform. A 10-kb SMRTbell library was prepared from sheared genomic DNA using a 10-kb template library preparation workflow. P4 chemistry was utilized, and the prepared library was sequenced on four single-molecule real-time (SMRT) cells, yielding output data with an average genome coverage of 148.61×. *De novo* assembly of the insert reads was performed with the Hierarchical Genome Assembly Process (HGAP) algorithm in SMRT Portal (version 2.1.1), in which the genome sequence of *Pandoraea* sp. strain RB-44 was assembled into a GC-rich (64.9%) single contig of 5,385,152 bp. rRNA and tRNA predictions were performed using ARAGORN (10) and RNAmmer (11), respectively, and the results revealed the presence of 69 tRNA genes and 12 rRNA operons in the genome.

Gene prediction was conducted using Prodigal version 2.60 (12), with which 4,781 open reading frames (ORFs) were predicted. Functional annotation of the predicted genes was performed by Blast2GO (13), which involved Gene Ontology (GO), enzyme code annotation with KEGG maps, and InterPro annotation. The predicted ORFs were also further annotated with an NCBI-NR comparison in which a LuxI homologue synthase and a LuxR homologue receptor were found adjacent to each other. The complete genome of *Pandoraea* sp. RB-44 is important for providing insight into the quorum-sensing activity of this soil bacterium.

### Nucleotide sequence accession numbers.

This complete genome project has been deposited in DDBJ/ENA/GenBank under the accession no. CP006938; the version described in this paper is the first version, CP006938.1.
